# Fungal Aerosol Exposure and Stage-Specific Variations in Taihang Chicken Houses During Winter

**DOI:** 10.3390/microorganisms13122856

**Published:** 2025-12-16

**Authors:** Yejin Yang, Zitong Yang, Zhibin Ren, Wenhao Feng, Zhenyue Li, Yi Lu, Mengxi Yan, Zhuhua Liu, Ran Zhu, Mingli Liu, Xiaolong Chen, Cheng Zhang, Shishan Dong, Huan Cui, Huage Liu

**Affiliations:** 1 The Animal Husbandry and Veterinary Institute of Hebei, Baoding 071001, China; 2 College of Veterinary Medicine, Hebei Agricultural University, Baoding 071000, China; 3 College of Medicine, Yanbian University, Yanji 133002, China

**Keywords:** fungal bioaerosols, Taihang chicken, particle size distribution, One Health, occupational exposure, poultry housing

## Abstract

Fungal aerosols are critical yet understudied bioaerosol components in enclosed poultry environments, particularly during winter when ventilation is restricted. This study investigated stage-specific variations in fungal aerosol concentration, size distribution, and community composition in Taihang chicken houses across three growth stages (15, 60, and 150 days). Culturable fungal concentrations significantly increased from 3.16 × 10^3^ CFU/m^3^ to 1.24 × 10^4^ CFU/m^3^ with bird age (*p* < 0.001, ANOVA). Respirable particles (<4.7 μm) consistently dominated the fungal size distribution. ITS sequencing revealed progressive increases in fungal richness and distinct community shifts among stages. Several fungi with zoonotic potential, including *Aspergillus*, *Cladosporium*, *Cryptococcus*, and *Fusarium*, were detected across all stages. These findings demonstrate that wintertime enclosed ventilation, while thermally beneficial, promotes the accumulation of respirable fungal aerosols and elevates occupational and environmental health risks. From a One Health perspective, stage-specific mitigation strategies—such as ventilation optimization, reduction in organic dust sources, and routine monitoring of respirable fungal fractions—are essential for reducing exposure risks in cold-season poultry production.

## 1. Introduction

Microbial aerosols represent one of the most epidemiologically significant pollutants in intensive poultry farming environments. They act as vectors for the transmission of fungi, bacteria, viruses, and their toxic metabolites, thereby establishing a potential respiratory exposure pathway between animals and farm workers. The rapid expansion of large-scale, intensive farming has led to a substantial increase in emissions of particulate matter (PM) and microorganisms within poultry houses. The resulting accumulation of complex bioaerosols has emerged as a critical factor threatening poultry health, compromising production performance, and impairing ambient air quality [1]. Animal-derived PM often carries fungal spores, endotoxins, antibiotic resistance genes, and heavy metal residues. These components can undergo long-distance transmission and subsequently induce respiratory tract irritation, allergic inflammation, and infections [1]. Studies have confirmed that poultry farms are significant emission sources of airborne fungi, with the inhalation of high spore concentrations being closely associated with asthma, allergic alveolitis, chronic cough, and other respiratory disorders in workers [2]. Consequently, fungal aerosols are not only a threat to animal health and productivity but also a quintessential One Health risk factor.

These risks are further amplified under winter production conditions in cold climates. To maintain thermal insulation and reduce energy consumption, poultry houses in northern China often adopt enclosed management practices with reduced ventilation rates. However, this leads to unintended consequences, including weakened PM settlement, prolonged suspension times, and the enrichment of fungal spores [3]. Driven by the combination of stable temperature/humidity conditions and a high organic load (e.g., feather dander, feed dust, and feces), winter poultry houses become an “ideal habitat” for fungal proliferation and spore dispersal. The resulting spore particles are predominantly within the respirable size range, possessing the potential for deep alveolar deposition [2]. Previous studies conducted in North American and European commercial hen houses have frequently detected dominant airborne fungi such as *Aspergillus*, *Cladosporium*, and *Penicillium* [4,5,6]. Some of these genera have been confirmed to be pathogenic, allergenic, or capable of producing mycotoxins, posing long-term exposure risks to poultry, workers, and nearby residents. Nevertheless, existing research has primarily focused on seasonal variations or regional monitoring, leaving a systematic understanding of the “stage-specific fungal risks” that evolve with poultry age notably lacking. Given the WHO’s recent Fungal Priority Pathogens List highlighting fungal pathogens as emerging global public-health threats, targeted investigation of fungal aerosols in intensive poultry houses is increasingly important [7,8].

The Taihang chicken is a representative indigenous breed in Hebei Province, valued for its robustness, adaptability to coarse feed, and excellent meat and egg quality. Its industry has undergone rapid expansion in recent years, fueled by consumer market upgrades [9,10,11]. As farming practices shift from traditional free-range systems to high-density, intensive cage systems [12], the pressures related to environmental control and health management have intensified. Significant differences in feed intake, metabolic intensity, feather shedding, manure excretion, and cage density across different growth stages inevitably lead to dynamic succession characteristics in the respective aerial microenvironments. However, critical knowledge gaps remain regarding the concentration dynamics, size distribution, and community succession of fungal aerosols across different age stages in enclosed Taihang chicken houses during winter. There is a particular scarcity of evidence directly linking particle size spectra to respiratory deposition risks and exposure to pathogenic fungi.

Therefore, this study employed a winter-housed, caged Taihang chicken farm as a model to investigate three key growth stages: brooding (15 days), growing (60 days), and laying (150 days). Utilizing an Andersen six-stage impactor coupled with culture-based quantification and ITS high-throughput sequencing, we systematically analyzed the concentration variations, particle size distribution dynamics, and community succession patterns of fungal aerosols. The potential zoonotic and occupational exposure risks were also assessed. This research aims to elucidate the stage-specific respiratory hazard characteristics of fungal aerosols, thereby providing a scientific basis for optimizing ventilation strategies, enhancing aerial biosecurity, protecting occupational health, and informing risk management within a One Health framework for poultry production in cold regions.

## 2. Materials and Methods

### 2.1. Study Sites, Season, and Experimental Design

The study was conducted in December 2024 across five large-scale and geographically dispersed Taihang chicken farms in Hebei Province, all operating under identical management protocols and belonging to the same company. All poultry houses featured fully enclosed structures equipped with mechanical ventilation and belt manure removal systems. During winter, ventilation rates were deliberately restricted to maintain a stable thermal environment, resulting in an overall air velocity below 0.20 m/s within the houses. Under these typical winter conditions, the houses were characterized by warmth, stable humidity, and high concentrations of organic dust. While this environment reduced heat loss, it also provided favorable conditions for the dispersal and accumulation of fungal spores.

To investigate the stage-specific risks of fungal aerosols, synchronized sampling was performed during three distinct growth phases: the brooding stage (15 days), the growing stage (60 days), and the laying stage (150 days). These stages represent critical points in the typical production cycle of Taihang chickens, marked by significant differences in physiological metabolism, manure output, feather shedding rate, and activity levels. The environmental parameters for each stage were as follows: Brooding stage (15 days): Temperature 30–35 °C, Relative Humidity 60–70%. Growing stage (60 days): Temperature 22–27 °C, Relative Humidity 55–65%. Laying stage (150 days): Temperature 15–20 °C, Relative Humidity 50–60%.

Within each house and for each stage, five fixed sampling points were established. These points were distributed across typical airflow paths at the front, middle, and rear sections of the houses, covering both upper and lower breathing zone heights to ensure the collected samples represented the overall airborne exposure profile. All sampling was completed on the same day, yielding a total of seventy-five original air samples. Five sampling points × five farms = 25 samples per stage, Three stages = 75 original samples in total. For the five samples collected per stage, a subset was used for fungal culture and counting to determine statistical differences in concentration and size distribution. The remaining samples were pooled by stage for subsequent ITS high-throughput sequencing to analyze the fungal aerosol community structure.

### 2.2. Airborne Fungal Sampling and Culturable Counting

Airborne fungal particles were collected using an Andersen six-stage cascade impactor, which effectively segregates and deposits airborne particles onto agar plates based on their aerodynamic diameter. The Andersen six-stage impactor was used exclusively for culturable concentration and particle-size distribution analyses. Sampling was conducted at a height of approximately 1.2–1.5 m above the ground, corresponding to the breathing zone of the chickens. The flow rate was set at 28.3 L/min, with a total sampled air volume of 300 L per sample. Following collection, the agar plates were immediately sealed for transport to minimize spore loss and environmental contamination.

Fungal isolation and cultivation were performed using Sabouraud dextrose agar. Plates were incubated at 28 °C for 5–7 days before colony counting. The final concentration of culturable fungi was calculated using the positive hole correction method [13]. Transport blank and field blank controls were included in each sampling batch to account for potential background contamination and ensure data reliability.

### 2.3. Aerosol Biomass Recovery and DNA Extraction

To further analyze the fungal community composition, aerosol biomass was collected using a high-volume air sampler (Model HH02-LS120, Beijing Huarui Hean Technology Co., Ltd., Beijing, China). The high-volume air sampler was used solely for biomass collection for ITS sequencing. The sampler was operated continuously for 12 h to enhance the capture efficiency of lower-concentration fungal spores. Sampling was performed using pre-combusted Tissuquartz™ quartz fiber filters (20.32 cm × 25.4 cm, PALL, Duncan, SC, USA) to minimize environmental DNA interference. After sampling, the filters were immediately stored at −80 °C. For nucleic acid extraction, the filters were cut into pieces, followed by steps of elution, centrifugation, and precipitation to collect the biomass. The resulting precipitate was used to extract total fungal genomic DNA using the cetyltrimethylammonium bromide (CTAB) method. DNA was extracted using a modified CTAB protocol that involved sequential cell lysis, chloroform–isoamyl alcohol extraction, and isopropanol precipitation to ensure efficient nucleic acid recovery [14]. The purity and integrity of the extracted DNA were verified by spectrophotometry and gel electrophoresis; samples failing quality checks were re-extracted to ensure the quality of subsequent amplification and sequencing. The Andersen impactor and the high-volume air sampler were deployed at the same sampling locations but served distinct analytical purposes. The former quantified culturable fungal concentrations and size distributions, whereas the latter collected sufficient aerosol biomass for ITS sequencing. The datasets generated by the two systems were therefore not directly compared or integrated. Because the Andersen impactor (≈300 L over 10.6 min) and the high-volume sampler (~12 h) capture aerosols over vastly different air volumes and temporal intervals, their outputs represent different temporal snapshots and were not intended for direct quantitative comparison. The two systems were used for complementary objectives rather than integrated analyses.

### 2.4. ITS Amplification, Library Preparation, Sequencing, and Bioinformatic Analysis

Following established protocols, the ITS1 region was targeted for PCR amplification to analyze the fungal community [15]. After amplification, the PCR products were purified by gel extraction and used to construct sequencing libraries. These libraries were then subjected to paired-end sequencing on an Illumina platform. The resulting raw sequences were processed through quality control, chimera removal, Operational Taxonomic Unit (OTU) clustering, and taxonomic annotation. The OTU clustering was performed at a 97% similarity threshold, and taxonomic annotation was conducted using the UNITE v9.0 (17 October 2022 release) database. The processed data were used for subsequent analyses of alpha diversity, beta diversity, and community succession patterns, and to generate visualizations of dominant fungal genera. The sequencing data have been deposited in the NCBI GenBank database under BioProject accession number PRJNA1354579.

### 2.5. Statistical Analysis

All data were statistically analyzed using SPSS software (version 28.0). Differences between groups were assessed by one-way analysis of variance (ANOVA), with a *p* < 0.05 considered statistically significant. When ANOVA identified significant differences, Tukey’s HSD test was applied for post hoc multiple comparisons. Differences in β-diversity among stages were evaluated using PERMANOVA (999 permutations) based on Bray–Curtis dissimilarity. The final results are presented as mean ± standard deviation.

## 3. Results

### 3.1. Concentration Variations and Size Distribution of Culturable Airborne Fungi

**Figure 1:**
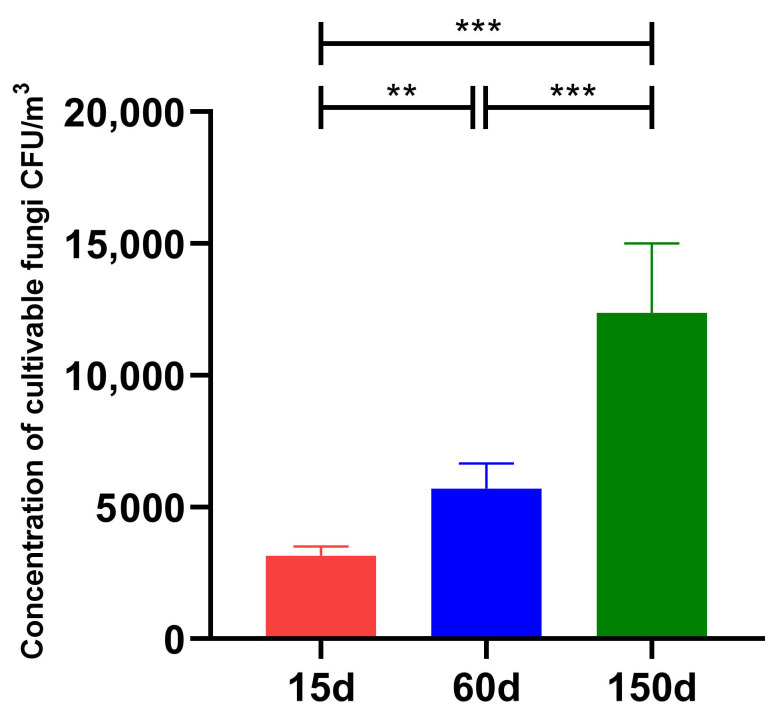
Concentrationsof culturable fungi in the air of Taihang chicken houses across different growth stages. The fungal concentration increased significantly with bird age (*p* < 0.001), demonstrating a clear stage-specific accumulation trend. ** *p* < 0.01, *** *p* < 0.001.

Significant differences were observed in the concentrations of culturable airborne fungi across the different growth stages (Figure 1). During the brooding stage (15 days), the fungal concentration was 3.16 × 10^3^ ± 0.36 × 10^3^ CFU/m^3^. This concentration increased significantly to 5.70 × 10^3^ ± 0.97 × 10^3^ CFU/m^3^ (*p* < 0.01) in the growing stage (60 days), and further rose to 1.24 × 10^4^ ± 2.64 × 10^3^ CFU/m^3^ (*p* < 0.001) in the laying stage (150 days). Furthermore, the concentration at 150 days was significantly higher than that at 60 days (*p* < 0.001). This trend indicates a clear age-dependent accumulation of fungal load in the enclosed winter environment. This phenomenon is presumably associated with increased host dander shedding, manure accumulation, rising organic dust concentrations, and enhanced activity leading to feather disturbance, which collectively promote the continuous emission and resuspension of aerosolized fungi. These findings align with recent reports on poultry bioaerosols [16].

**Figure 2:**
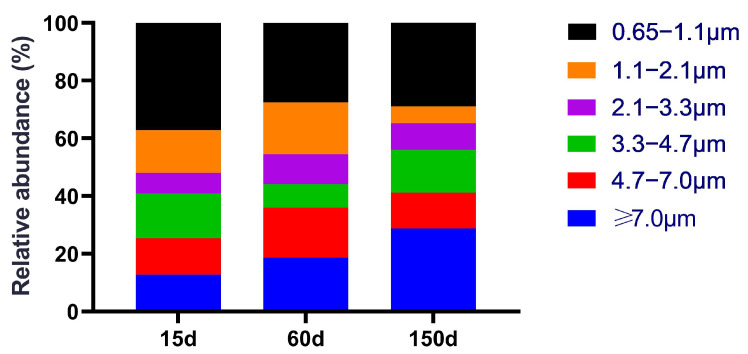
Size distribution of fungal aerosols in the air of Taihang chicken houses. Respirable spores (<4.7 μm) constituted the dominant proportion across all stages. This proportion was highest during the brooding stage and exhibited a slight shift towards larger particles as the birds aged.

Andersen cascade impactor results revealed distinct differences in the size distribution of fungal aerosols across the stages (Figure 2). In the 15-day samples, small-sized fungal aerosols (<4.7 μm) constituted the highest proportion (76.79%), with ultrafine inhalable particles (0.65–1.1 μm) being the most dominant fraction (38.26%). This indicates that airborne fungal spores during the brooding stage are highly suspendable and inhalable, and present a high risk of deep lung deposition. By 60 days, the proportion of small particles decreased to 64.05%, suggesting that a greater fraction of fungi tended to attach to larger particle carriers, facilitating enhanced sedimentation. At 150 days, the size distribution shifted further towards larger particles (>4.7 μm accounted for 41.46%), yet nearly 60% of spores remained in the small particle fraction. This dynamic suggests that deeply respirable fungi constituted a significant proportion across all three stages. Their size characteristics correspond to aerodynamic properties that allow them to bypass upper respiratory defenses and deposit in the alveolar region, thereby significantly elevating the respiratory exposure risk for both farm workers and the flock [17]. Overall, the fungal concentration increased with age, while the fungal aerosol was predominantly composed of respirable spores, indicating a persistent potential for respiratory hazards throughout the production cycle in winter-housed Taihang chicken houses.

### 3.2. Sequencing Results and OTU Richness Variations in the Fungal Community

**Figure 3:**
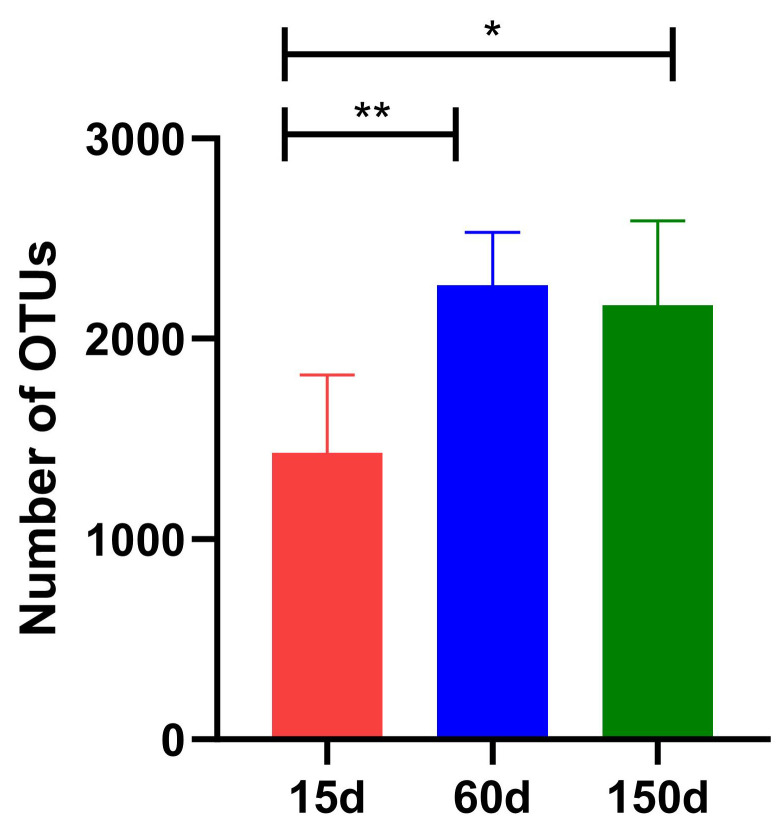
Operational Taxonomic Unit (OTU) numbers of the airborne fungal communities in Taihang chicken houses at different stages. The OTU number increased significantly with bird age (*p* < 0.01), reflecting a cumulative expansion of the fungal community. * *p* < 0.05, ** *p* < 0.01.

After quality control, a total of 1.11 × 10^4^ high-quality sequences were obtained for analysis. The number of Operational Taxonomic Unit (OTU) showed a significant positive correlation with bird age (Figure 3). The OTU count was 1.43 × 10^3^ ± 0.39 × 10^3^ at 15 days, increased significantly to 2.27 × 10^3^ ± 0.27 × 10^3^ at 60 days (*p* < 0.01), and reached 2.17 × 10^3^ ± 0.42 × 10^3^ at 150 days (*p* < 0.01). Although OTU richness at 150 days was significantly higher than at 15 days (*p* < 0.01), the difference between 60 and 150 days was not statistically significant (*p* > 0.05). These results demonstrate a cumulative expansion of the fungal community within the enclosed winter environment as the chickens aged, indicating that the sources and dispersal of fungi are influenced by host growth activity and environmental organic load.

### 3.3. Differences in Fungal Community Alpha and Beta Diversity

**Figure 4:**
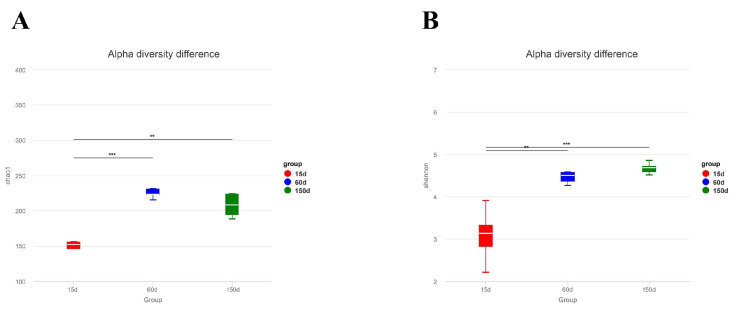
Alpha diversityindices of the airborne fungal communities in Taihang chicken houses. Both the (**A**) Chao1 and (**B**) Shannon indices increased significantly with bird age (*p* < 0.01), indicating a progressive enhancement in fungal community richness and evenness. ** *p* < 0.01, *** *p* < 0.001.

Alpha diversity analysis revealed significant differences in the fungal communities across the growth stages (Figure 4). The Chao1 index increased from 145.77 ± 40.30 at 15 days to 233.03 ± 24.70 at 60 days (*p* < 0.001), remaining at a high level of 223.41 ± 43.79 at 150 days (*p* < 0.01). Similarly, the Shannon index significantly increased from 3.08 ± 0.62 to 4.54 ± 0.29 at 60 days (*p* < 0.01) and further to 4.67 ± 0.13 at 150 days (*p* < 0.001). These results indicate significant age-dependent increases in both species richness and community evenness, with the most pronounced expansion occurring between 15 and 60 days of age.

**Figure 5:**
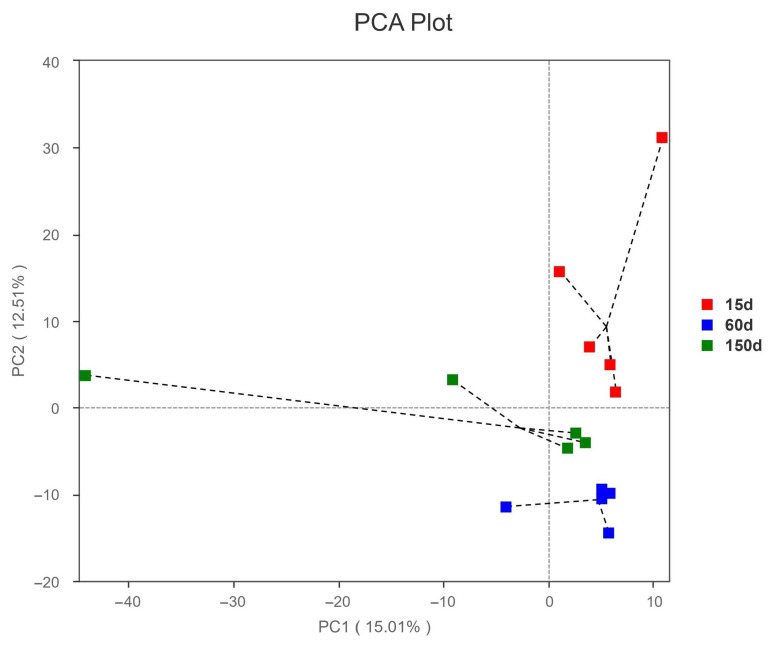
Principal component analysis (PCA) of beta diversity for the airborne fungal communities in Taihang chicken houses. Clear separation among samples from different age groups was observed, suggesting distinct, stage-specific differentiation in fungal community structure.

Principal Components Analysis (PCA) of beta diversity showed clear separation among the samples from the three stages (Figure 5), indicating that the fungal community structure was strongly driven by host age and exhibited stage-specific niche differentiation. This divergence trend may be associated with changes in stocking density, manure output, accumulation of organic dust, variations in aerosol carrier properties, and differences in ventilation intensity.

### 3.4. Fungal Community Composition and Potential Zoonotic Risks

**Figure 6:**
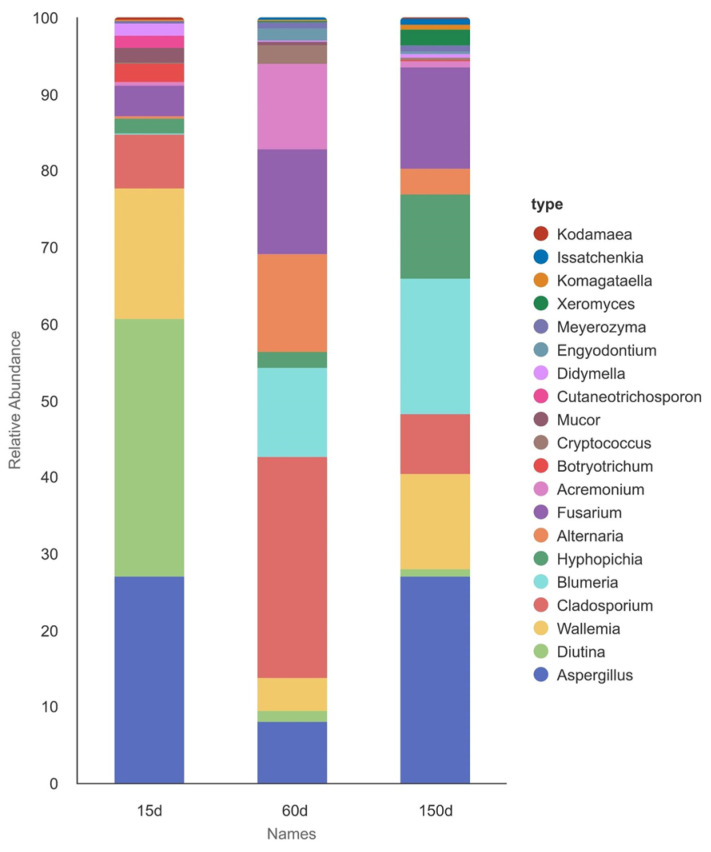
Composition of the dominant genera in the airborne fungal communities of Taihang chicken houses across different growth stages. The fungal community exhibited a clear successional trend. The genus *Aspergillus* maintained a high relative abundance throughout all stages.

**Figure 7:**
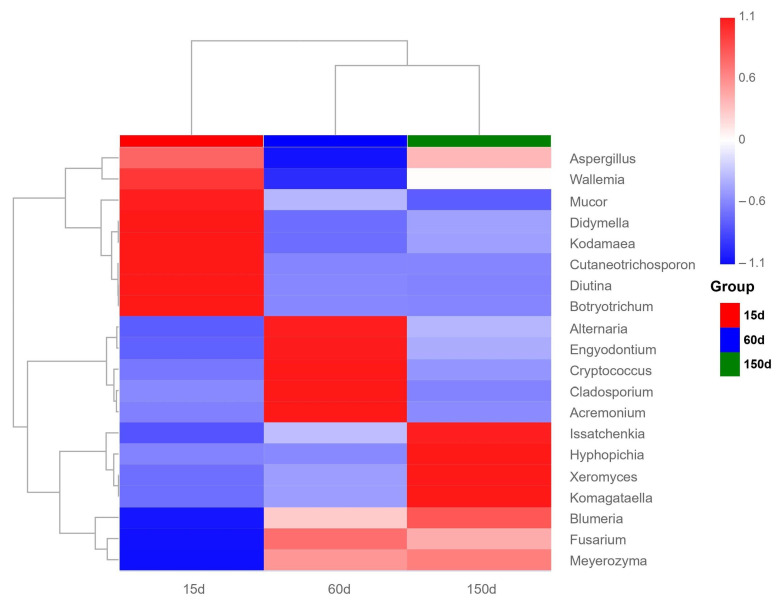
Relative abundance of potentially zoonotic fungi detected in the air of Taihang chicken houses. The detection of genera such as *Aspergillus*, *Cladosporium*, *Fusarium*, and *Cryptococcus* indicates potential occupational exposure risks in the enclosed poultry houses during winter.

Analysis of dominant fungal genera revealed significant successional changes across the different age stages (Figure 6 and Figure 7). The relative abundances of *Blumeria*, *Hyphopichia*, *Xeromyces*, and *Komagataella* peaked at 150 days. In contrast, fungi such as *Diutina* and *Mucor* were dominant at 15 days and subsequently declined with age. Genera including *Cladosporium*, *Alternaria*, *Fusarium*, *Acremonium*, and *Cryptococcus* exhibited enrichment during the intermediate stage. Notably, *Aspergillus* maintained a high relative abundance across all three stages, indicating its strong environmental competitiveness throughout the entire production cycle, which aligns closely with previous studies [18].

It is particularly noteworthy that several fungi closely associated with zoonotic risks were detected, including *Aspergillus*, *Cladosporium*, *Cryptococcus*, and *Fusarium*. To complement the relative abundance profiles, Supplementary Table S1 provides the absolute read counts and prevalence of major pathogenic genera, offering a more comprehensive assessment of their potential exposure risks. Among these, *Aspergillus fumigatus* and *Cryptococcus* neoformans are globally significant pathogenic species capable of causing pulmonary invasive fungal diseases, exacerbation of asthma, or central nervous system infections [19,20,21]. *Fusarium* species can produce mycotoxins and induce respiratory symptoms [22]. These findings suggest that the enclosed poultry house environment in winter not only impacts flock health but also poses occupational exposure risks and public health concerns for farm workers.

## 4. Discussion

Airborne bioaerosols are a significant component of the environment in intensive animal husbandry, acting as key vectors for particulate matter (PM), microorganisms, and their bioactive constituents, often coexisting with gaseous pollutants like ammonia [2,6,23]. Poultry farms are recognized as point sources of high-concentration bioaerosols, which can impair poultry productivity and pose respiratory health risks to workers. In recent years, the World Health Organization (WHO) has further underscored the public health threat of airborne fungi and the rising trend of antifungal resistance through its “Fungal Priority Pathogens List,” highlighting the urgency of enhanced monitoring and intervention at the human–animal-environment interface [1,7,8].

This study observed a significant increase in culturable fungal concentrations across the growth stages (from 3.16 × 10^3^ to 1.24 × 10^4^ CFU/m^3^) in winter-housed, caged Taihang chicken houses. This finding aligns with numerous reports from poultry facilities in Canada, Europe, and West Asia, where total airborne fungi in layer and broiler houses typically range from 10^3^ to 10^4^ CFU/m^3^ and show a consistent upward trend as the rearing cycle progresses, particularly during low-ventilation seasons [1,4,24]. This consistent trend across climatic regions can be mechanistically explained by a triad of factors: (i) insufficient dilution and removal due to minimal winter ventilation, (ii) continuous substrate supply from litter, feather debris, feed dust, and manure under high-density rearing, and (iii) stable temperature and humidity conditions that favor spore viability [2,25]. This scenario creates a cumulative exposure profile, consistent with winter confinement practices, indicating the need for differentiated ventilation and end-of-pipe purification strategies for poultry houses in cold regions during seasonal transitions.

Our results demonstrated that the fungal particle size distribution was skewed towards, or persistently dominated by, the respirable fraction (<4.7 μm) across all three stages. Particle size is a critical determinant of deposition site and subsequent health outcomes. Particles ≥ 4.7 μm predominantly deposit in the upper airways, inducing mucosal irritation and local inflammation. In contrast, spores and fragments < 4.7 μm can bypass mucociliary clearance and deposit in the alveoli, triggering oxidative stress and neutrophilic inflammatory responses characterized by IL-1β/IL-8 release, which are associated with phenotypes like occupational asthma [23,26,27]. Poultry studies from Europe and North America have similarly reported correlations between a high proportion of respirable particles and respiratory symptoms, supporting the use of the “respirable fraction” as a more sensitive exposure metric than total concentration [24,28,29]. Consequently, the combination of “stage-dependent increase” and “respirable fraction dominance” constitutes a classic breathing hazard scenario in winter poultry houses, warranting priority in monitoring and intervention efforts. Importantly, these stage-specific trends provide actionable guidance for risk mitigation. The brooding and growing stages—when respirable particles and sensitive fungal genera peak—represent critical windows for strengthening ventilation management, minimizing organic dust accumulation, and implementing targeted cleaning schedules. Additionally, our data support adopting respirable-fraction–focused monitoring rather than relying solely on total fungal concentration, as this more accurately reflects exposure hazards for both birds and workers.

ITS sequencing revealed a significant increase in alpha diversity with bird age and clear stage-specific separation in beta diversity, accompanied by successional changes in dominant genera. This aligns with recent poultry house studies from the EU, South America, and East Asia, indicating that the fungal community is not mere “background noise” but a dynamic ecosystem driven by substrate release, resuspension, and host status [24,30,31,32]. Previous research has indicated a significant homology between fungal communities in poultry manure and air, with activities like manure removal facilitating their aerosolization [24,33,34]. Our stage-specific data further corroborate the ecological continuum of “manure–air–host,” explaining the diversity surge and genus-level restructuring observed during the growing stage (60 days). The dominant or frequently detected genera in this study, such as *Aspergillus*, *Cladosporium*, *Fusarium*, and *Cryptococcus*, are highly consistent with findings from recent surveys of poultry air and litter in Canada, Europe, and China [4,16,17,21,24,35]. *Aspergillus fumigatus* is linked to allergic bronchopulmonary aspergillosis and invasive aspergillosis, with its small, highly respirable spores posing a significant risk. *Cladosporium* and *Alternaria* are consistently associated with asthma exacerbation and allergic reactions. *Fusarium* constitutes a “dual-channel” exposure risk via both the feed chain and the airborne route. *Cryptococcus* exhibits high pathogenicity in immunocompromised hosts [1,19,21,36,37]. This combination of “dominant respirable size” and “the presence of zoonotic/allergenic species” corresponds well with the respiratory symptoms and lung function decline observed among poultry workers in occupational health studies across multiple countries [38,39]. This underscores the need to concurrently assess risks for susceptible populations and high-exposure job roles during winter confinement. From a One Health perspective, the detection of potentially zoonotic fungi reinforces the need for integrated management strategies that simultaneously address animal health (e.g., maintaining dry manure), human occupational safety (e.g., improved personal protective equipment and dust control), and environmental stewardship (e.g., reducing pathogen drift through controlled ventilation and filtration).

Beyond the winter confinement context of this study, other research points to seasonal variations in fungal communities, with factors like *Aspergillus* being more common in summer and *Mucor* and *Penicillium* more prominent in autumn/winter [40]. These dynamics are influenced by the interplay of temperature/humidity windows, ventilation strategies, and litter moisture content. Comparative studies in Canadian layer farms have shown differences in dust/bioaerosol metrics between conventional and alternative housing systems, yet ventilation and end-of-line filtration remain common “leverage points” determining exposure levels in both systems [24]. Our results are consistent with these studies in principle, identifying ventilation management and material management (e.g., maintaining dry litter, enclosed manure transfer) as primary measures for mitigating peak exposures in winter.

Real-world poultry house exposure represents a complex mixture of “fungi × bacteria × endotoxins × β-glucans × ammonia” [23]. Multiple assessments suggest that co-exposure to fungal Pathogen-Associated Molecular Patterns, such as β-glucans, and endotoxins can synergistically amplify the NF-κB pathway and pro-inflammatory cytokine release, suggesting that health risks may be underestimated [41]. Furthermore, increasing reports of azole-resistant *Aspergillus* in agricultural and livestock environments highlight the emergence of an antifungal resistance ecology from a One Health perspective, necessitating its inclusion, alongside operational safety, in risk governance frameworks [1,13]. Although poultry-house air contains a complex mixture of biological and particulate hazards, including bacteria, viruses, endotoxins, and organic dust—this study specifically evaluates the fungal component of that exposure matrix. As such, the risk patterns identified here should be interpreted as one facet of a multifactorial airborne environment, highlighting the need for integrated assessments that consider multiple hazard categories in future research.

This study has several limitations that should be considered when interpreting the findings, many of which also highlight important directions for future research. First, to obtain sufficient biomass for ITS sequencing, samples from the five farms were pooled by growth stage. Although this approach increased DNA yield, it limited the ability to assess inter-farm variability. Future work should incorporate farm-specific sequencing to capture finer spatial heterogeneity and improve ecological resolution. Second, sampling at each growth stage was conducted on a single day, providing a cross-sectional rather than longitudinal perspective. Repeated or continuous monitoring within the same houses would enable more accurate characterization of temporal succession and short-term aerosol dynamics. Third, all sampling occurred during winter under enclosed ventilation conditions. Because ventilation patterns, temperature, and humidity differ markedly across seasons, year-round sampling campaigns are needed to determine whether the patterns observed here persist under warmer, open-ventilation conditions. Fourth, the culture-based and sequencing-based datasets were generated using sampling systems with vastly different air volumes and temporal integration (≈10 min for the Andersen impactor vs. 12 h for the high-volume sampler). These differences limit direct correspondence between culturable concentrations and ITS-derived community profiles. Future studies should consider harmonized sampling durations or parallel time-resolved sampling to better integrate viability-based and sequence-based assessments. Finally, culture-based methods inherently detect only viable fungi, while ITS sequencing does not differentiate between viable and non-viable spores. Future efforts incorporating viability assays, RNA-based activity markers, or shotgun metagenomics will provide a more comprehensive understanding of exposure risk. Although ITS sequencing cannot detect antifungal resistance markers, our findings underscore the importance of surveillance for antifungal resistance within agricultural bioaerosols. Integrating ITS profiling with shotgun metagenomics in future work will enable identification of resistance-associated genes and support proactive One Health strategies. Overall, by linking stage-specific fungal exposure patterns with feasible pathways for methodological improvement, these limitations highlight clear research priorities for developing more accurate, integrated, and actionable airborne risk assessments in poultry production systems.

## 5. Conclusions

This study demonstrates that fungal aerosols in enclosed Taihang chicken houses show distinct stage-specific patterns during winter, marked by increasing concentrations, dominance of respirable particles (<4.7 μm), and shifts in community composition. The presence of fungi with zoonotic potential—including *Aspergillus*, *Cladosporium*, *Fusarium*, and *Cryptococcus*—indicates meaningful exposure risks for both poultry and workers. From a One Health perspective, winter management strategies should focus on reducing respirable fungal exposure through improved ventilation, source control, and routine airborne monitoring. Future research integrating year-round sampling and multi-omics approaches will be essential for developing evidence-based, stage-tailored mitigation frameworks for sustainable poultry production in cold climates.

## Data Availability

The data presented in this study are openly available in the NCBI BioProject database at https://www.ncbi.nlm.nih.gov/bioproject/?term=PRJNA1354579, reference number PRJNA1354579 (accessed on 8 December 2025).

## Supplementary Materials

The following supporting information can be downloaded at: https://www.mdpi.com/article/10.3390/microorganisms13122856/s1, Table S1: Absolute read counts and prevalence of major potentially pathogenic fungal genera detected in airborne fungal communities at 15, 60, and 150 days.

## References

[1] Yan H.L., Li Y., Zhang Y., Zhang H.F., Guo Z.D., Liu J.B. (2021). Deciphering of microbial diversity and antibiotic resistome of bioaerosols in swine confinement buildings. Sci. Total Environ..

[2] Sowiak M., Bródka K., Kozajda A., Buczyńska A., Szadkowska-Stańczyk I. (2012). Fungal aerosol in the process of poultry breeding—Quantitative and qualitative analysis. Med. Pr..

[3] Wang Y., Li X., Geng H., Zhu Z., Wang Q., Dong H. (2023). Variation of PM2.5 and PM10 in emissions and chemical compositions in different seasons from a manure-belt laying hen house. Poult. Sci..

[4] St-Germain M.W., Létourneau V., Larios Martínez A.D., Godbout S., Boulianne M., Duchaine C. (2024). Airborne dust and bioaerosols in Canadian conventional and alternative houses for laying hens. J. Occup. Environ. Hyg..

[5] Górny R.L., Gołofit-Szymczak M., Cyprowski M., Ławniczek-Wałczyk A., Stobnicka A., Wolska L.A. (2023). Poultry house as point source of intense bioaerosol emission. Ann. Agric. Environ. Med..

[6] Almatawah Q.A., Al-Khalaifah H.S., Aldameer A.S., Ali A.K., Benhaji A.H., Varghese J.S. (2023). Microbiological indoor and outdoor air quality in chicken fattening houses. J. Environ. Public Health.

[7] Casalini G., Giacomelli A., Antinori S. (2024). The WHO fungal priority pathogens list: A crucial reappraisal to review the prioritisation. Lancet Microbe.

[8] Beardsley J. (2024). Pathogens of importance in lung disease—Implications of the WHO fungal priority pathogen list. Respirology.

[9] Han H., Sun Y., Fan Y., Zhang H., Yang J., Chi R., Gao Y., Liu J., Li K., Li W. (2022). Microbial diversity and community composition of duodenum microbiota of high and low egg-yielding Taihang chickens identified using 16S rRNA amplicon sequencing. Life.

[10] Fan Y., Wu X., Li Y., Han H., Zhang Y., Yang J., Liu Y. (2022). Effect of polymorphisms in the 5′-flanking sequence of MC1R on feather color in Taihang chickens. Poult. Sci..

[11] Chen H., Wu X., Cui S., Li Y., Mu Y., Gao J., Liu H., Liu J. (2024). Residue elimination patterns and determination of the withdrawal times of seven antibiotics in eggs of Taihang chickens. Animals.

[12] Qiaoxian Y., Hui C., Yingjue X., Chenxuan H., Jianzhong X., Rongyan Z., Lijun X., Han W., Ye C. (2020). Effect of housing system and age on products and bone properties of Taihang chickens. Poult. Sci..

[13] Wang X., Cui H., Li Z., Yang Z., Liu H., Wang J., Zhang N., Li J., Chen X., Zhang C. (2025). Distribution of aerosol bacteria in broiler houses at different growth stages during winter. Animals.

[14] Doyle J.J., Doyle J.L. (1987). A rapid DNA isolation procedure for small amounts of fresh leaf tissue. Plant Mol. Biol. Rep..

[15] Chen Y., Liang Z., Li G., An T. (2024). Indoor/outdoor airborne microbiome characteristics in residential areas across four seasons and its indoor purification. Environ. Int..

[16] Chen G., Ma D., Huang Q., Tang W., Wei M., Li Y., Jiang L., Zhu H., Yu X., Zheng W. (2021). Aerosol concentrations and fungal communities within broiler houses in different broiler growth stages in summer. Front. Vet. Sci..

[17] Byrd J.A., Caldwell D.Y., Nisbet D.J. (2017). The identification of fungi collected from the ceca of commercial poultry. Poult. Sci..

[18] Xu C.-L., Wang C., Li G.-B., Zhao T., Zhou R.-L., Chen J.J. (2024). Antibiotic administration aggravates asthma by disrupting gut microbiota and intestinal mucosal barrier in an asthma mouse model. Environ. Med..

[19] Earle K., Valero C., Conn D.P., Vere G., Cook P.C., Bromley M.J., Bowyer P., Gago S. (2023). Pathogenicity and virulence of Aspergillus fumigatus. Virulence.

[20] Olsen Y., Arildskov E., Hansen S.N., Pedersen M., Dharmage S.C., Kloster M., Sigsgaard T. (2023). Outdoor Alternaria and Cladosporium spores and acute asthma. Clin. Exp. Allergy.

[21] Francisco E.C., de Jong A.W., Hagen F. (2021). Cryptococcosis and Cryptococcus. Mycopathologia.

[22] Niculita-Hirzel H., Hantier G., Storti F., Plateel G., Roger T. (2016). Frequent occupational exposure to Fusarium mycotoxins of workers in the Swiss grain industry. Toxins.

[23] Pan Y., Zhang W., Xu Z., Zuo Z., Yuan T. (2024). Fungal community shows more variations by season and particle size than bacteria. Sci. Total Environ..

[24] Wang R., Yu A., Qiu T., Guo Y., Gao H., Sun X., Gao M., Wang X. (2022). Aerosolization behaviour of fungi and its potential health effects during the composting of animal manure. Int. J. Environ. Res. Public Health.

[25] St-Germain M.W., Létourneau V., Cruaud P., Lemaille C., Robitaille K., Denis É., Boulianne M., Duchaine C. (2024). Longitudinal survey of total airborne bacterial and archaeal concentrations and bacterial diversity in enriched colony housing and aviaries for laying hens. Poult. Sci..

[26] Xie X., Wang P., Jin M., Wang Y., Qi L., Wu C., Guo S., Li C., Zhang X., Yuan Y. (2024). IL-1β-induced epithelial cell and fibroblast transdifferentiation promotes neutrophil recruitment in chronic rhinosinusitis with nasal polyps. Nat. Commun..

[27] Park H.S., Jung K.S., Hwang S.C., Nahm D.H., Yim H.E. (1998). Neutrophil infiltration and release of IL-8 in airway mucosa from subjects with grain-dust-induced occupational asthma. Clin. Exp. Allergy.

[28] Viegas S., Faísca V.M., Dias H., Clérigo A., Carolino E., Viegas C. (2013). Occupational exposure to poultry dust and effects on the respiratory system in workers. J. Toxicol. Environ. Health A.

[29] Kearney G.D., Shaw R., Prentice M., Tutor-Marcom R. (2014). Evaluation of respiratory symptoms and respiratory protection behavior among poultry workers in small farming operations. J. Agromed..

[30] Robinson K., Yang Q., Stewart S., Whitmore M.A., Zhang G. (2022). Biogeography, succession, and origin of the chicken intestinal mycobiome. Microbiome.

[31] Viegas C., Carolino E., Malta-Vacas J., Sabino R., Viegas S., Veríssimo C. (2012). Fungal contamination of poultry litter: A public health problem. J. Toxicol. Environ. Health A.

[32] Rodrigues Marcondes N., Ledesma Taira C., Cirena Vandresen D., Estivalet Svidzinski T.I., Kadowaki M.K., Peralta R.M. (2008). New feather-degrading filamentous fungi. Microb. Ecol..

[33] Gontar Ł., Sitarek-Andrzejczyk M., Kochański M., Buła M., Drutowska A., Zych D., Markiewicz J. (2022). Dynamics and diversity of microbial contamination in poultry bedding materials containing medicinal plant components. Materials.

[34] Kubasova T., Faldynova M., Crhanova M., Karasova D., Zeman M., Babak V., Rychlik I. (2022). Succession, replacement, and modification of chicken litter microbiota. Appl. Environ. Microbiol..

[35] Gomes B., Pena P., Cervantes R., Dias M., Viegas C. (2022). Microbial contamination of bedding material: One Health in poultry production. Int. J. Environ. Res. Public Health.

[36] Kulcsár S., Turbók J., Kövér G., Balogh K., Zándoki E., Ali O., Szabó A., Mézes M. (2024). Exposure to a combination of Fusarium mycotoxins leads to lipid peroxidation and influences antioxidant defenses, fatty-acid composition of phospholipids, and renal histology in laying hens. Toxins.

[37] Zhao Y., Lin X. (2021). Cryptococcus neoformans: Sex, morphogenesis, and virulence. Infect. Genet. Evol..

[38] Skóra J., Matusiak K., Wojewódzki P., Nowak A., Sulyok M., Ligocka A., Okrasa M., Hermann J., Gutarowska B. (2016). Evaluation of microbiological and chemical contaminants in poultry farms. Int. J. Environ. Res. Public Health.

[39] Ngajilo D., Singh T., Ratshikhopha E., Dayal P., Matuka O., Baatjies R., Jeebhay M.F. (2018). Risk factors associated with allergic sensitization and asthma phenotypes among poultry farm workers. Am. J. Ind. Med..

[40] Ostović M., Ravić I., Kovačić M., Ekert Kabalin A., Matković K., Sabolek I., Pavičić Ž., Menčik S., Horvatek Tomić D. (2021). Differences in fungal contamination of broiler litter between summer and winter fattening periods. Arch. Ind. Hyg. Toxicol..

[41] McBride M.A., Stothers C.L., Fensterheim B.A., Caja K.R., Owen A.M., Hernandez A., Bohannon J.K., Patil N.K., Ali S., Dalal S. (2024). Bacteria- and fungus-derived PAMPs induce innate immune memory via similar functional, metabolic, and transcriptional adaptations. J. Leukoc. Biol..

