# Supplementary material for: Fungal Aerosol Exposure and Stage-Specific Variations in Taihang Chicken Houses During Winter
Source: Microorganisms. 2025 Dec 16;13(12):2856. doi: 10.3390/microorganisms13122856 (PMC12736169; doi:10.3390/microorganisms13122856)
Supplement: Supplementary file 1 [file microorganisms-13-02856-s001.zip › Supplementary Materials.pdf]

Supplementary Table S1. Absolute read counts and prevalence of major potentially pathogenic fungal genera detected in airborne fungal communities at 15, 60, and 150 days.

Genus	15 days — Absolute Reads	15 days — Prevalence (%)	60 days — Absolute Reads	60 days — Prevalence (%)	150 days — Absolute Reads	150 days — Prevalence (%)
Aspergillus	1,742	100%	4,368	100%	3,921	100%
Cladosporium	287	80%	1,412	100%	1,176	100%
Fusarium	203	60%	1,015	80%	894	80%
Cryptococcus	138	40%	587	80%	653	80%
Alternaria	76	20%	455	80%	402	80%
Acremonium	63	20%	274	40%	251	40%
Mucor	223	60%	104	20%	57	20%
Diutina	247	60%	121	40%	66	20%
